# Prevalence and pattern of sexual assault in Usmanu Danfodiyo University Teaching Hospital, Sokoto, Nigeria

**DOI:** 10.11604/pamj.2016.24.332.9462

**Published:** 2016-08-30

**Authors:** Mairo Hassan, Kehinde Joseph Awosan, Abubakar Abubakar Panti, Sadiya Nasir, Karima Tunau, Amina Gambo Umar, Constance Egondu Shehu, Aeron Eze Ukwu, Bilal Sulaiman

**Affiliations:** 1 Department of Obstetrics and Gynecology, Usmanu Danfodiyo University Teaching Hospital, Sokoto, Nigeria; 2 Department of Community Medicine, Usmanu Danfodiyo University Teaching Hospital, Sokoto, Nigeria

**Keywords:** Prevalence, pattern, sexual assault, UDUTH, Sokoto

## Abstract

**Introduction:**

Sexual violence is an important public health problem of growing concern all over the world. This study was conducted to determine the prevalence and pattern of sexual assault managed in Usmanu Danfodiyo University Teaching Hospital Sokoto, Nigeria.

**Methods:**

It was a retrospective study that looked into cases of sexual assault admitted into the hospital between January 2010 and December 2014. Information on patients’ biodata, and relevant details on the cases were extracted from the patients’ case files and analyzed.

**Results:**

Out of the 5317 gynecological admissions during the period under study, 45 (0.84%) were cases of sexual assault. Of these, only 34 case files were available for data extraction. The patients’ ages ranged from 2 to 37 years (mean = 12.6 + 8.3). About two thirds (61.8%) of those affected were young children (aged 12 years and below). In majority of cases (70.6%) the assault was penetrative, and in most of the cases (91.2%) only a single assailant was involved. In close to two thirds of cases, the assailant was either an acquaintance (38.2%) or a family member (20.6%). Although law enforcement agents were informed in majority (58.8%) of cases, arrests were made in less than half (41.2%).

**Conclusion:**

Although the prevalence of sexual assault in this study appears to be low, a major cause for concern is the fact that those affected were predominantly young children. Parents should be more vigilant in monitoring their children’s movement, and stringent laws should be enacted and enforced to curb this heinous act.

## Introduction

Sexual violence is an important public health problem of growing concern all over the world. Sexual abuse is an unwanted sexual activity in which the perpetrator uses force, threats or takes advantage of victim not able to give consent. When the force used is immediate, of short duration or infrequent it is termed as sexual assault. The offender is referred to as sexual abuser, or molester [1]. The term also covers any behavior by an adult towards a child to stimulate either the adult or the child sexually. When the victim is younger than age of consent, it is referred to as child sexual abuse. Both sexes are affected and most victims and perpetrators know each other. The assailants usually range from family members to acquaintances to strangers [2–5]. Sexual abuse is associated with significant morbidity and mortality. Various types of injuries are seen as a result of physical force such as severe multiple bruises in uncommon sites, vaginal and anal lacerations and perforations have been reported [6–10]. The victim is also exposed to sexually-transmitted disease, psychological trauma and risk of unwanted pregnancy [11, 12]. Although the trauma of the assault heals with time, it leaves long term psychological and medical problems behind [13]. Younger juvenile victims are more likely than older victims to be sexually abused [14, 15]. The abuse may involve use of objects, forcible fondling, and forcible sodomy. Forcible rape has been shown to be more likely to involve a single victim than any other sexual assault. Personal weapons such as hands, fist, and legs are commonly used during rape however other weapons such as knife and gun have been noted to be used. Records have shown that in some cases no weapon was used (only verbal threats), but the use of weapons is more likely when the victim is older [16, 17]. The time of the day when sexual abuse occurs is related primarily to the age of the victim. For adult victims sexual assault is more likely to occur after midnight while the pattern in juvenile assault is said to be earlier in the day (during or after school hours) [18, 19]. Nearly all the offenders reported to the law enforcement agents were males. However, female molesters commonly assault children under the age of 6 years. Juvenile assaults were more likely to result in arrests by law enforcement agents [20, 21].

In the United States, nearly 1 in 5 (18.3%) women and 1 in 75 (1.4%) men reported experiencing rape at some time in their lives [22]. Similar findings were obtained in studies across Africa. In a study in the Democratic Republic of Congo, 16% of women reported having sex against their will [23]. In another study among female university students in Ethiopia, 14.3% reported having experienced completed rape since being admitted into the university [24]. Also, in a study in South Africa, 24.9% of young men reported having raped a female previously [25]. In Nigeria, whereas relatively low prevalence of sexual assault was obtained in most of the health facility based studies ranging from 0.06% in Zaria [21], 0.76% in Lagos [15], 2.1% in Calabar [18], to 5.2% of all gynecological emergencies in Ile-Ife [8], a very high prevalence of sexual assuault was reported in community based studies across the country ranging from 14% among out of school adolescents in an urban slum in Lagos [16], 69.9% among juvenile female street hawkers from two urban towns in Anambra state [17] to 78.5% among employed girls in Maiduguri [14]. Despite the legal provisions of life jail with or without canning for sexual assaulters in Nigeria [26], the high prevalence of sexual assault in community based studies across the country may not be unconnected with the fact that most cases of rape are unreported by the victims out of fear of stigmatization, rejection by the society, and safety concerns, coupled with the fact that even for cases that are reported, the perpetrators are rarely prosecuted. Other forces believed to be responsible for the high prevalence of sexual assault in Nigerian include the enduring culture of male dominance, female social and economic disempowerment, and lack of synergy in civil society initiatives [27]. A study like this has never been conducted in Sokoto, the prevalence of sexual assault in Sokoto is therefore unknown despite the fact that sexual assaults exist in this locality. This study was therefore conducted to determine the prevalence and pattern of sexual assault seen at the Usmanu Danfodiyo University Teaching Hospital, Sokoto, Nigeria, with a view to making recommendations towards addressing the problem in the study area.

## Methods

This was a retrospective study based on data extracted from the case files of patients seen at the Usmanu Danfodiyo University Teaching Hospital (UDUTH), Sokoto, North-western Nigeria, between January 2012 and December 2014. The hospital is a referral centre that caters for Sokoto, Zamfara and Kebbi states. The hospital offers preventive, promotive and curative services. Case files of all patients presenting with a history of sexual assault were reviewed. A designed proforma was used to extract information on patient’s bio data, type of assault, mode of presentation, type(s) of injury sustained, weapon(s) of assault, relationship of offender to the victim, and involvement of law enforcement agents. Data cleansing was done, and then analyzed using IBM SPSS version 20 statistical software package, and the results were presented as descriptive and inferential statistics. All levels of statistical significance were set at p < 0.05. Ethical approval was obtained from the Ethical Committee of the hospital.

## Results

### Prevalence of sexual assault and socio-demographic profile of patients

There were 5317 gynecological cases seen during the period under review out of which 45 were cases of sexual assault, giving a prevalence of 0.84%. Out of the 45 cases of sexual assault seen at the hospital in the period under review, only 34 case files were available for data extraction. Almost all, 33 (97.1%) of the 34 patients were females. The patients’ ages ranged from 2 to 37 years (Mean = 12.6 ± 8.3). A larger proportion of victims (41.2%) aged 5 to 12 years; and about a fifth of victims (20.6%) were preschool children aged 4 years and below. Young children aged 12 years and below therefore accounted for majority (61.8%) of victims as shown in Table 1.

**Table 1:** Socio-demographic profile of patients

Variables	Frequency (%), n = 34
**Age groups (in years)**	
0 - 4	7 (20.6)
5 - 12	14 (41.2)
13 - 19	7 (20.6)
20 - 39	6 (17.6)
**Sex**	
Male	1 (2.9)
Female	33 (97.1)

### Place and type of assault

Majority of the assaults took place either in the homes (38.2%) or neighborhood (14.7%) of the victims or assaulters. Four (11.8%) of the 34 victims were assaulted in the class room. There was penetration of the vagina in majority, 24 (70.6%) of the 34 patients (mostly penile or fingering by the assailants) as shown in Table 2. Vaginal penetration was significantly more prevalent among victims in the 5-12 years age group (92.9%), while fondling was more prevalent among those aged 4 years and below (85.7%), Fisher’s Exact χ^2^ = 12.942, p = 0.002.

**Table 2:** Place and type of assault

Variables	Frequency (%), n = 34
**Place of assault**	
Home	13 (38.2)
Neighbourhood	5 (14.7)
Not documented	5 (14.7)
Class room	4 (11.8)
Shop	2 (5.8)
Bush	2 (5.8)
Road side	2 (5.8)
**Type of assault**	
Penetrative	24 (70.6)
Non-penetrative	10 (29.4)

### Mode of presentation and type of injury sustained

The most common mode of presentation was vaginal bleeding (50.0%), while the most common injury sustained was vaginal laceration (some of which necessitated examination under anesthesia and suturing of the laceration in order to arrest the bleeding). One death was recorded (a 9 year old girl brought in dead with her throat cut, and with bleeding from major vessels). Other modes of presentation and types of injury sustained are as shown in Table 3.

### Time interval between occurrence of assault and presentation

The time interval between occurrence of assault and presentation at the hospital ranged from less than 1 hour to 168 hours (median = 11 hours). Although, majority of the victims (44.1%) presented at the hospital within 8 hours following the assault, about a fifth (20.9%) of victims presented more than 24 hours after the assault (**Table 4**).

**Table 3:** Mode of presentation and type of injury sustained

Variables	Frequency (%), n = 34
**Mode of presentation**	
Vaginal bleeding	17 (50.0)
Secretions on the vulva	4 (11.8)
Perineal swelling	7 (20.5)
Vaginal discharge	2 (5.9)
Brought in dead	1 (2.9)
Acute urinary retention	1 (2.9)
Neck laceration	1 (2.9)
Facial swelling	1 (2.9)
**Type of injury sustained**	
Vaginal laceration	10 (29.4)
Perineal bruises/lacerations	9 (26.4)
Vaginal bruises	7 (20.5)
No demonstrable injury	7 (20.5)
Neck laceration	1 (2.9)

**Table 4:** Time interval between occurrence of assault and presentation

Time interval (in hours)	Frequency (%), n = 34
0 – 8	15 (44.1)
9 – 16	6 (17.6)
17 – 24	1 (2.9)
> 24	7 (20.9)
Not documented	5 (14.7)

### Use of weapon in assault

No weapon was used to subdue the victims by the assailants in most, 15 (44.1%) of the 34 cases seen. Fourteen (41.1%) were threatened verbally, while weapons were used in the remaining 5 (14.7%) cases, of which knife was used in 4 and gun was used in the remaining one (Table 5). Although no weapon was used in cases of victims aged 4 years and below, there was no significant association between age of victim and use of weapon (Fisher's Exact χ^2^ = 13.618, p = 0.623).

**Table 5:** Use of weapons in assault

Weapon used	Frequency (%), n = 34
None	15 (44.1)
Verbal threats	14 (41.1
Knife	4 (11.8)
Gun	1 (2.9)

### Profile of assailants

In majority, 31 (91.2%) of the 34 cases of assault, only 1 assailant was involved. In more than half, 20 (58.8%) of cases the assaulters were known to the victim (either acquaintance or family member). The occupation of most of the assailants was not known in most cases (Table 6).

**Table 6:** Profile of assailants

Variables	Frequency (%), n = 34
**Number of assailants**	
1	31 (91.2)
2	2 (5.8)
11	1 (2.9)
**Nature of assailants**	
Stranger	14 (41.2)
Acquaintance	13 (38.2)
Family member	7 (20.6)
**Occupation of assailants**	
Not documented	10 (29.0)
Civil servant	4 (11.6)
Student	5 (14.5)
Trader	4 (11.6)
Unemployed	9 (26.1)
Artisan	2 (5.8)

### Involvement of law enforcement agents

Although reports were made to the law enforcement agents in majority 20 (58.8%) of the 34 cases, the assailants were arrested in less than half 14 (41.7%) of the cases (Table 7).

**Table 7:** Involvement of law enforcement agents

Variables	Frequency (%), n = 34
**Case reported to law enforcement agents**	
Yes	20 (58.8)
No	9 (26.5)
Not documented	5 (14.7)
**Arrest made**	
Yes	14 (41.2)
No	15 (44.1)
Not documented	5 (14.7)

## Discussion

Although the prevalence of sexual assault was relatively low (0.84%) in this study, it confirms the existence of the phenomenon in Sokoto, Nigeria. This figure probably represents the tip of the iceberg in terms of its burden in the study area, if the low prevalence of sexual assault reported in health facilities based studies [15, 18, 21] is juxtaposed with the very high prevalence reported in community based studies [14, 16, 17] across Nigeria. It is important to note that the prevalence of sexual assault recorded in this study is much higher than that obtained in a study in Zaria, also located in north western Nigeria that reported a prevalence of 0.06% [21]. While studies conducted in Lagos and Ile-lfe that reported prevalence of 0.76% [15] and 0.69% [8] respectively, compare well with the finding in this study, other health facilities based studies by Ashimi et al, in a rural tertiary health facility in north western Nigeria [11] and Daru et al, in Jos [28] reported higher prevalence of 3.0% and 5.6% respectively. These findings show that sexual assault is incontrovertibly a problem in Sokoto, just like other parts of Nigeria. About two-thirds (61.8%) of the victims in this study were young children aged 12 years and below. This is in agreement with the findings in studies by Bhattacharyya et al [29] and Lakew [30] in which teenagers accounted for majority of cases. The assault took place in either the victims or assailants homes in more than half of cases (54%), and in two-thirds of cases the assailants were either acquaintances (38.2%) or family members (20.6%). These findings are in concordance with the findings in studies conducted in Benin [31] and Osogbo [19]. This is not surprising considering the vulnerability of the age group involved. Most of the victims were children, who were possibly left in the care of these family members or neighbors in the absence of their parents.

Vaginal bleeding due to laceration was the commonest mode of presentation seen in this study (29.4%). This is similar to the finding in a study conducted in Birnin-Kudu, North-West Nigeria, where vaginal laceration constituted 24% of cases [11]. A possible explanation for these findings is that the two studies were conducted within the same geopolitical zone of Nigeria, perhaps with similar socio-cultural factors. However, in contrast to the finding in this study where only a fifth (20.5%) of victims presented with no demonstrable injury, majority (70.8%) of the cases in the Birni-Kudu study had no demonstrable injury. Majority of the victims in this study (61.7%) presented within 8 to 16 hours of the assault. This is in contrast to the findings from Lagos [15] and Benin City [31], where 64.5% and 47% respectively presented late. Only a few (14.7%) victims were assaulted with weapons in this study, this could be due to the fact that they were mostly young children, and were less likely to struggle with the assailants to warrant the use of weapons. Also, majority of the victims (91.2%) were assaulted by only one assailant, who was also known to them in majority of cases. This is similar to the finding in other studies [8, 32] that reported that majority of victims were assaulted by person(s) known to them. One of the most interesting findings of this study is the fact that majority of cases (58.8%) were reported to law enforcement agents, even though arrests were made in less than half (41.2%) of cases. This may not be unconnected with the fact that most of the victims were young children and their parents could have taken the decision to report the incident on their behalf, unlike adult victims that would have concealed the incident and suffer in silence due to fear of stigmatization, rejection by the society and safety concerns. This finding agrees with the findings in other studies [20, 21] that reported higher likelihood of arrests by law enforcement agents in juvenile assaults.

## Conclusion

Although the prevalence of sexual assault in this study appears to be low, it confirms the existence of the phenomenon in Sokoto, Nigeria, and a major cause for concern is the fact that those affected were predominantly young children. Parents should be more vigilant in monitoring their children’s movements, and stringent laws should be enacted and enforced to curb this heinous act.

### What is known about this topic

- Sexual assault is prevalent across Nigeria, similar to the situation across the world;
- Majority of victims are often aged less than 18 years and the assaults are mostly perpetrated by persons known to them;
- Majority of cases go unreported due to fear of stigmatization, rejection by the society and safety concerns.

### What this study adds

- Sexual assault exists in Sokoto, Nigeria, even though the prevalence appears to be low;
- Victims were predominantly young children aged 12 years and below;
- Whereas, law enforcement agents were informed in most of the cases, arrests were made in less than half.
